# Postoperative Drip-Infusion of Remifentanil Reduces Postoperative Pain—A Retrospective Observative Study

**DOI:** 10.3390/ijerph18179225

**Published:** 2021-09-01

**Authors:** Yi-Hsuan Huang, Meei-Shyuan Lee, Yao-Tsung Lin, Nian-Cih Huang, Jing Kao, Hou-Chuan Lai, Bo-Feng Lin, Kuang-I Cheng, Zhi-Fu Wu

**Affiliations:** 1 Department of Anesthesiology, Tri-Service General Hospital, National Defense Medical Center, Taipei 11490, Taiwan; yixiun72@gmail.com (Y.-H.H.); niancih@hotmail.com (N.-C.H.); m99ane@gmail.com (H.-C.L.); bflin0114@gmail.com (B.-F.L.); 2 School of Public Health, National Defense Medical Center, Taipei 11490, Taiwan; mmsl@mail.ndmctsgh.edu.tw (M.-S.L.); kosukeluvu@gmail.com (J.K.); 3 Department of Anesthesiology, Chi Mei Medical Center, Tainan 71004, Taiwan; anekevin@hotmail.com; 4 Department of Food Science and Technology, Chia Nan University of Pharmacy and Science, Tainan 71710, Taiwan; 5 Department of Anesthesiology, Kaohsiung Medical University Hospital, Kaohsiung Medical University, Kaohsiung 80756, Taiwan; kuaich@gmail.com; 6 Department of Anesthesiology, Faculty of Medicine, College of Medicine, Kaohsiung Medical University, Kaohsiung 80708, Taiwan

**Keywords:** remifentanil, hyperalgesia, drip infusion, gradual withdrawal

## Abstract

Development of remifentanil-induced hyperalgesia (RIH) postoperatively is an unpleasant experience that requires further treatment. This study assessed the effects of gradual withdrawal combined with drip infusion of remifentanil on postoperative pain and the requirement for rescue analgesics. A total of 559 patients receiving total intravenous anesthesia with propofol and remifentanil were enrolled. All patients either underwent gradual withdrawal of remifentanil (GWR) or gradual withdrawal combined with drip infusion (GWDR) with a dose of 1 mcg·kg^−1^ for 30 min after extubation. The numeric rating scale (NRS) and the requirement of rescue analgesics were assessed. The requirement for rescue analgesics was significantly lower in the GWDR group than in the GWR group (13.2% vs. 35.7%; *p* < 0.001). At the post-anesthetic care unit (PACU), patients in the GWDR group had a lower NRS pain score (*p* < 0.001). In addition, in the postoperative 2nd hour, patients in the GWDR group had a significantly lower NRS than the GWR group (beta, −0.31; *p* = 0.003). No remifentanil-related adverse effects were observed. We found that gradual withdrawal combined with drip infusion of remifentanil required less rescue analgesics and reduced pain scores. The new way of remifentanil administration may be effective to prevent RIH.

## 1. Introduction

Opioids are a major component of analgesia in the clinical practice of anesthesia. Among them, the application of ultra-short-acting opioids, remifentanil, with its unique chemical structure and high lipid affinity, offers the potent and rapid-onset properties and plays a key role in total intravenous anesthesia (TIVA) [1]. Consequently, the combination of remifentanil and propofol by target-controlled infusion (TCI) was the so-called “ideal” TIVA [2] to accomplish adequate analgesia, depth of anesthesia, and rapid postoperative recovery.

The intraoperative use of short-acting opioids is associated with opioid-induced hyperalgesia (OIH) after surgery [3]. Once early prevention of OIH is established perioperatively, delayed recovery/discharge and further development of chronic pain can be avoided. As a result, the concern of OIH has risen in the perioperative period, particularly remifentanil, a short-acting opioid [4].

Strategies aimed at mitigating remifentanil-induced hyperalgesia (RIH) have been reported, including the use of the lowest dosage of remifentanil, multimodal analgesia, and alternative management, such as another opioid and gradual withdrawal of remifentanil (GWR) [5].

A randomized, double-blinded, placebo-controlled, crossover trial included 19 healthy volunteers and suggested that the GWR could reduce RIH in the heat pain test [6]. In addition, Saxena et al. [7] conducted a double-blinded, randomized controlled study and concluded that gradual withdrawal of remifentanil after thyroid surgery may delay the initial postoperative requirement of analgesics, but the overall consumption of opioids, pain scores, Ramsay Sedation Scale scores, and quality of recovery scores (QoR-40) were similar in both abrupt discontinuation and gradual withdrawal groups. Nonetheless, the withdrawal process requires vigilance and training. In clinical practice, GWR during surgery is unsatisfactory. To the best of our knowledge, only one case report described that drip infusion of remifentanil after surgery could attenuate postoperative pain in our institute [8]. We conducted a single-centre retrospective cohort study to assess GWR and drip infusion of remifentanil immediately after extubation affected postoperative pain score and the use of analgesics.

## 2. Materials and Methods

### 2.1. Study Design

This was a retrospective cohort study.

### 2.2. Setting

This study was conducted at the Tri-Service General Hospital (Taipei, Taiwan).

### 2.3. Participants and Data Sources

After approval from the ethics committee (TSGHIRB No: 2-108-05-135) of Tri-Service General Hospital (TSGH), Taipei, Taiwan, relevant information was retrieved from the medical records and the electronic database of TSGH, and the requirement for written informed consent was waived by the IRB. This retrospective study included 559 American Society of Anesthesiologists (ASA) scores of I–III patients receiving surgery under TIVA with propofol and remifentanil from May 2018 to January 2019 without receiving postoperative patient-controlled analgesia. Three hundred and fifty-four patients were subjected to TIVA without remifentanil dripped (Group GWR) after extubation and 205 underwent surgery under the influence of drip infusion of remifentanil (Group GWDR) after extubation. No inhalation anesthesia, a combination of propofol and inhalation anesthesia, or regional anesthesia were used in these patients. The exclusion criteria were the use of inhalation anesthesia or propofol combined with inhalation anesthesia, regional anesthesia, pregnancy, previous substance abuse, a neuropsychiatric disorder, or age <20 years or >80 years.

### 2.4. Anesthesia and Perioperative Management

No prior medications were prescribed before the induction of anesthesia. Standard hemodynamic monitoring, including non-invasive blood pressure, electrocardiography (lead II), pulse oximetry, and end-tidal carbon dioxide pressure, were performed. Anesthesia was induced by intravenous propofol and remifentanil via a TCI pump (Fresenius Orchestra Primea; Fresenius Kabi AG, Germany) and lidocaine (2%, 1.5 mg·kg^−1^) in all patients. Tracheal intubation was facilitated with rocuronium 0.6–0.8 mg·kg^−1^. Anesthesia was maintained using concentration effect-site (Ce) of propofol with Schnider pharmacokinetic model ranged between 2.0–2.5 mcg·mL^−1^ and of remifentanil with Minto pharmacokinetic model ranged between 1.5–3.0 ng·mL^−1^ and a fresh gas flow of 0.3 L·min^−1^ of oxygen and air with inspired oxygen fraction 0.5. Repetitive bolus dosing of rocuronium (0.2 mg·kg^−1^) was administered as required during the return of neuromuscular function. The Ce of propofol and remifentanil was adjusted in the range of 0.2–0.5 mcg·mL^−1^ and 0.2–0.5 ng·mL^−1^, respectively, with necessary according to the hemodynamic changes and bispectral index (BIS) monitoring (with a BIS value of 40–60). End-tidal carbon dioxide pressure was maintained at 35–45 mmHg by modulating the ventilation rate and maximum airway pressure. Only patients undergoing thoracic surgery received an intraoperative intercostal nerve block. Thirty minutes before skin closure, ketorolac 30 mg was administered intravenously. At 10–20 min before the end of the surgery, patients were prescribed with the Ce of remifentanil tapering to 1 ng·mL^−1^. At the end of the surgery, propofol was discontinued while patients were ventilated with 100% oxygen at a fresh gas flow rate of 6 L·min^−1^ [8]. The endotracheal tube was removed after successful reversal of rocuronium and ensuring patients’ responses to verbal commands with adequate spontaneous and smooth breathing. Subsequently, remifentanil was tapered off, and the patients were transferred to the post-anesthetic care unit (PACU) for further care.

In the GWDR group, further withdrawal of remifentanil was administered by dripping 1 mcg·kg^−1^ of remifentanil in a volume-controlled burette with 50 mL normal saline for 30 min by the preference of the attending anesthesiologist after tracheal extubation immediately [8]. In the control group, no medication was administered after tracheal extubation.

In the PACU, the numeric rating scale (NRS) was assessed 30 min after extubation, and rescue analgesic, fentanyl 50 mcg, was administered if the patient’s pain was more than 3/10 of NRS or the patient requested due to intolerant pain. All patients were monitored under adequate surveillance, stayed at least 60 min in the PACU, and were discharged from the PACU toward the wards until NRS < 3/10 without nausea, vomiting, or any discomfort. In the ward, tramadol or NSAIDs were prescribed regularly for pain management and rescue analgesic with additional tramadol was prescribed if NRS ≥ 4 every 6 h. We also recorded NRS and adverse effects at 2 h after surgery.

### 2.5. Variables

The retrospectively collected patient data were obtained from the medical records and electronic database: age at the time of surgery, demographic characteristics, body mass index (BMI), ASA score, NRS scores in the PACU, postoperative rescue analgesics, anesthesia time, surgical time, and perioperative consumption of propofol and remifentanil. To differentiate the effects of drip infusion of remifentanil on the degrees of pain, patients were classified into three pain categories by NRS scores, namely no pain (0), mild pain (1–3/10), and moderate to severe pain (≥4/10) which rescue analgesics were required [7]. In addition, we recorded the types of surgeries that were divided into major and minor surgeries, and surgical sites.

### 2.6. Study Sample Size

Based on our institute data, a sample size analysis was performed with the incidence of rescue analgesics in PACU being 36 % after TIVA as the primary variable. To achieve a power of 90% at a two-tailed type I error of 0.05, at least 189 patients in each group were required to detect 40% (reduced from 36% to 21%) of the intergroup difference.

### 2.7. Statistical Analysis

The major aim of our study was to identify the effectiveness of drip infusion of remifentanil on postoperative pain. The primary outcome was the comparison of the requirement for postoperative rescue analgesics during PACU between the two groups. The secondary outcomes were NRS scores 30 min after extubation in the PACU and 2 h after surgery in the ward, the observation of adverse effects, the type of surgery, surgical site, and anesthetic consumption, which may influence the development of RIH.

Data are presented as mean ± standard deviation or number (percentage) of patients. Continuous variables were compared using the Student *t*-test or Mann-Whitney U test if the data were not distributed normally. Categorical variables were compared using the chi-square or Fisher’s exact test, when appropriate. Multinominal logistic regression analyses were used to evaluate the relationship between drip infusion of remifentanil and the degrees of pain (mild or moderate to severe pain vs. no pain), as well as rescue analgesics. In the multivariable models, variables were entered by forward selection to avoid multicollinearity. Estimates of odds ratios (ORs) and associated 95% confidence intervals (CIs) were obtained from these models. NRS was also treated as a continuous variable, and linear regression was used to assess the effect of postoperative drip infusion of remifentanil at the PACU as well as at the 2nd hour after surgery.

Statistical significance was accepted for a two-tailed *p*-value of less than 0.05. Statistical analysis was performed using SPSS version 23 statistical software program (SPSS Inc., Chicago, IL, USA).

## 3. Results

### 3.1. Baseline Characteristics

A total of 559 patients who underwent surgery were reviewed, of which 205 received drip infusion of remifentanil and 354 received no intravenous medication after surgery. Table 1 shows the baseline characteristics and intraoperative data of the patients. Patient demographics, including age and weight, and intraoperative remifentanil consumption, were similar in both groups. The percentage of male patients was higher in the GWDR group (45.4%) than in the GWR group (35.0%; *p* = 0.019). The surgical sites of the GWDR group were significantly more located on the skin and connective tissue, extremities, and chest than those of the GWR group (*p* < 0.001). The GWDR group had significantly more patients undergoing major surgery than the GWR group (49.3% vs. 33.1%; *p* < 0.001). The incidence of postoperative rescue analgesic requirement was lower in the GWDR group than in the GWR group (13.2% vs. 35.7%; *p* < 0.001). The GWDR group had a lower NRS score than GWR group (*p* < 0.001). There were only one and four patients in the GWR and GWDR groups, respectively, with NRS ≥ 7. There were too few patients with severe pain to be identified as the difference between the two groups. More propofol consumption was in the GWDR group than in the GWR group (85.1 ± 65.1 mL *vs.* 76.1 ± 47.8 mL; *p* < 0.001). No adverse effects, such as nausea, vomiting, hypotension, respiratory depression, requirement of oxygen or mechanical ventilation were found in the PACU and 2 h after surgery in both groups.

### 3.2. Variables Associated with NRS and Use of Rescue Analgesics according to Univariate and Multivariable Analysis

Table 2 shows the comparison of perioperative factors with postoperative degrees of pain. Multivariable analyses showed some variables related to mild or moderate to severe pain, including drip infusion of remifentanil, type of surgery, and surgical site. After multivariable analyses, patients in the GWDR group had a significantly lower risk of experiencing mild pain (1–3/10) than the GWR group (OR, 0.30; 95% CI, 0.17–0.55; *p* < 0.001). Patients undergoing minor surgery had a significantly lower risk of suffering from mild pain than patients undergoing major surgery (OR, 0.34; 95% CI, 0.15–0.79; *p* = 0.012). Significantly more patients whose surgical sites were located on the musculoskeletal and abdominal regions experienced mild pain than those whose surgical site was located on the skin and connective tissue (musculoskeletal: OR, 5.09; 95% CI, 2.14–12.1; *p* < 0.001 and abdomen: OR, 5.03; 95% CI, 2.30–11.0; *p* < 0.001). Similarly, patients in the GWDR group had a significantly lower risk of experiencing moderate to severe pain (≥4/10) than the GWR group (OR, 0.11; 95% CI, 0.05–0.24; *p* < 0.001). Patients undergoing minor surgery had a significantly lower risk of suffering from moderate to severe pain than patients undergoing major surgery (OR, 0.30; 95% CI, 0.11–0.80; *p* = 0.016). Significantly more patients whose surgical sites were located in the musculoskeletal, chest, and abdominal regions had moderate to severe pain than those whose surgical site was located on the skin and connective tissue (musculoskeletal: OR, 10.9; 95% CI, 3.66–32.2; *p* < 0.001; chest: OR, 4.88; 95% CI, 1.01–23.5; *p* = 0.048; abdomen: OR, 10.9; 95% CI, 3.88–30.4; *p* < 0.001, respectively).

Table 3 shows the comparisons of NRS at the PACU (30 min after extubation) and in the postoperative 2nd hour. Multivariable analyses revealed that not only patients in the GWDR group had a significantly lower NRS than the GWR group at the PACU (beta, −1.20; 95% CI, −1.51–0.89; *p* < 0.001), but also patients in the GWDR group had a significantly lower NRS in the postoperative 2nd hour than the GWR group (beta, −0.31; 95% CI, −0.52–−0.11; *p* = 0.003). Patients who underwent minor surgery had significantly lower NRS scores than those who underwent major surgery at the PACU (beta, −0.55; 95% CI −0.94–−0.16; *p* = 0.006). In addition, multivariable analysis showed significantly higher NRS in the postoperative 2nd hour in the abdominal surgery (*p* = 0.016) and female sex (*p* < 0.001). In the postoperative 2nd hour, there was no significant difference in NRS in both patients undergoing major and minor surgery after univariate analysis (beta, 0.06; 95% CI, −0.15–−0.27; *p* = 0.555).

Table 4 shows the comparison of perioperative factors with the use of rescue analgesics. Multivariable analyses showed some variables related to the use of rescue analgesics, including drip infusion of remifentanil, type of surgery, and surgical site. Patients in the GWDR group had significantly fewer requirements for rescue analgesics than the GWR group (OR, 0.14; 95% CI, 0.08–0.24; *p* < 0.001). Patients undergoing minor surgery had significantly fewer rescue analgesics than patients undergoing major surgery (OR, 0.43; 95% CI, 0.24–0.75; *p* = 0.003). Patients whose surgical sites were located in the musculoskeletal, chest, and abdominal regions required significantly more rescue analgesics than those whose surgical sites were located on the skin and connective tissue (musculoskeletal: OR, 3.24; 95% CI, 1.62–6.51; *p* = 0.001; chest: OR, 9.86; 95% CI, 3.41–28.5; *p* < 0.001; abdomen: OR, 4.27; 95% CI, 2.24–8.14; *p* < 0.001, respectively). Patients with higher body weight required fewer rescue analgesics than those with smaller body weight (OR, 0.84; 95% CI, 0.72–0.99; *p* = 0.035).

## 4. Discussion

Our retrospective study demonstrated that gradual withdrawal during surgery and drip infusion of remifentanil immediately after surgery effectively lowered the use of rescue analgesics as well as the NRS after 30 min at the PACU and the postoperative 2nd hour without respiratory depression. This result was consistent with previous reports [7,9] with continuous infusion of remifentanil persistently in the PACU after thyroid surgery and laparoscopic-assisted vaginal hysterectomy. In addition, we also suggested that drip infusion of remifentanil in major surgeries such as musculoskeletal, thoracic, and abdominal surgeries is necessary (Table 4).

The development of OIH debates and the underlying cellular and molecular mechanisms are complex and involve interactions between neurons, glial cells, transient receptor potential vanilloid channels, cytokines, neurokinin-1 receptors, serotonin receptor type 3, cholecystokinin, μ opioid receptor signaling, long-term potentiation (LTP), N-methyl-d-aspartate receptors, and other transcriptional mechanisms [3,10,11,12,13]. Treatment or prevention strategies basically originated from these mechanisms, with proven clinical evidence.

There are many strategies, such as propofol [14], ketamine [15], dexmedetomidine [16], nitrous oxide (N_2_O) [17], non-steroidal anti-inflammatory drugs (NSAIDs) [18,19], buprenorphine [20], etc., which are recognized to be effective in preventing RIH. One of the most interesting theories is opioid withdrawal LTP. Drdla et al. [21] investigated intravenous infusion of remifentanil with a bolus (30 mcg·kg^−1^) followed by an infusion (450 mcg·kg^−1^·hour^−1^ for 1 h) and tapered withdrawal of remifentanil for 30 min in vivo. They showed that withdrawal LTP may be prevented by tapering the remifentanil infusion instead of abrupt withdrawal through the mechanism of decrement of potentiation of spinal dorsal horn C-fiber-evoked field potentials in all five animals tested (*p* = 0.001). In brief, application of remifentanil in vivo leads to acute depression of synaptic strength in C-fibers; upon withdrawal, synaptic strength not only quickly returns to normal but becomes potentiated for prolonged periods of time [22]. Consequently, the GWR might be a potential modality for the prevention of RIH.

There are few studies on GWR in the prevention of RIH in clinical practice. Comelon et al. [6] described the administration of remifentanil at 2.5 ng·mL^−1^ for 30 min and gradual withdrawal by 0.6 ng·mL^−1^ every 5 min by TCI pump in healthy volunteers and concluded patients receiving GWR had significantly lower NRS scores without the development of RIH in heat pain but not cold test than those receiving the abrupt withdrawal. Saxena et al. [7] illustrated a reduction of remifentanil at an infusion rate of 30% every 15 minutes by a TCI pump from 2 to 0 ng·mL^−1^ 2 h after thyroid surgery and revealed a significantly delayed demand for postoperative analgesics but an insignificant decrease in pain scores with gradual withdrawal of remifentanil. Lee et al. [9] reported that continuous infusion of remifentanil 0.05 and 0.1 mcg·kg^−1^·min^−1^ immediately after laparoscopic-assisted vaginal hysterectomy for 30 min with alfentanil-based patient-controlled analgesia showed a similar effect on pain scores and respiratory depression. The analgesic effects of GWR (Ce, 1.0 ng·mL^−1^ and persisted for 15 min) in our study did not satisfy almost 36% of patients with requiring rescue analgesics. The result might be different from gradual withdrawal via 0.6 ng·mL^−1^ every 5 min for 15 min by Comelon et al. [6], who concluded effective prevention of RIH in heat pain test. The pain pathway from heat and surgery may differ, resulting in different consequences of our study from Comelon et al. [6]. Additionally, GWR was ineffective in the cold pain test, which involved a large skin surface area and was affected by vasomotor regulation [6] as well as major surgery. The incidence of postoperative rescue analgesic requirement in the GWDR group remained at 13.2%, implying that another analgesic strategy was necessary. The findings of our retrospective study assented to this point of view clinically by drip infusion of remifentanil as a bridge therapeutic strategy after continuous infusion of remifentanil during surgery and provided an alternative method of drip infusion of remifentanil rather than TCI or continuous infusion with the syringe pump of remifentanil [7,9,23,24,25], especially in institutes where TCI or syringe pumps are not available in the PACU, such as in our hospital. Indeed, an additional remifentanil drip-infusion may reduce pain scores and rescue analgesics. The analgesic effect of drip infusion of remifentanil might be minimal in the postoperative recovery period because the dosage of remifentanil was low (1.0 mcg·mL^−1^) and the infusion period was short (30 min). In addition, the context sensitive half-time of remifentanil was 3.4 min.

A previous systemic review and meta-analysis enrolled 1494 patients in 27 studies and found a significant increase in acute pain at 4 and 24 h postoperatively after high intraoperative consumption of remifentanil and consequently higher morphine requirement on postoperative day 1 [26]. Guignard et al. [27] demonstrated that patients undergoing major abdominal surgery with administration larger dosage of remifentanil (0.3 ± 0.2 mcg·kg^−1^·min^−1^) had significant higher visual analog pain scores and cumulative morphine consumption than those with administration lower dosage of remifentanil (0.1 ± 0.0 mcg·kg^−1^·min^−1^). Furthermore, administration of a cumulated dose greater than 50 mcg·kg^−1^ remifentanil intraoperatively would be associated with exacerbated postoperative pain score and/or multiplied opioid requisites [28], but all our patients received a cumulative dose of less than 50 mcg·kg^−1^. We also showed that patients who had longer surgical and anesthesia times and higher consumption of propofol had a higher NRS and requirement of postoperative rescue analgesics than those who were not. Undoubtedly, our patients who underwent major surgery had higher pain scores and needed more rescue analgesics postoperatively than those who underwent minor surgery. Meanwhile, our patients undergoing thoracic surgery had no significant difference in mild pain (*p* = 0.083) and a marginally significant difference in moderate to severe pain (*p* = 0.048) in comparison of patients undergoing surgery of skin and connective tissue (Table 2), which might have resulted from the intraoperative intercostal nerve block prescribed in patients undergoing thoracic surgery. We suggest that strategies to prevent RIH are crucial to improving recovery after major surgery, such as musculoskeletal, thoracic, and abdominal surgery.

Our study had some limitations. First, this was a retrospective study. Patients were not randomly allocated, and characteristics, such as ASA score, type of surgery, and surgical sites, may have introduced uncontrolled biases. We could neither guarantee that all patients received drip infusion of remifentanil with the same protocol, even though we had checked all the anesthetic documents, including records of anesthetic, and consumption of remifentanil in the PACU for assurance of the properly practiced protocol. Moreover, this alternative modality has been practiced for several years [8]. As a result, we believe the correct practice rate was high in our institute. Second, hyperalgesia was not measured by specific devices (von Frey filaments), or pressure threshold algometers [29]. Instead, the pain was recorded using the NRS and the consumption of rescue analgesics. Interestingly, Koppert et al. [30] investigated that RIH was related to significantly higher NRS and larger hyperalgesic area than control values. Although hyperalgesia was not measured in our patients, drip-infusion of remifentanil significantly reduced NRS which may give clinicians a hint that drip infusion of remifentanil could lower the development of RIH. In addition, regular analgesics, tramadol 100 mg every 8 h and ketorolac 30 mg every 8 h, were administered intravenously, and rescue analgesic with additional tramadol was prescribed intravenously if NRS ≥ 4 every 6 h, which may be one of the confounding factors of our results. Consequently, the alternative administration of remifentanil may encourage clinicians to reduce RIH in clinical practice. Third, we only observed patients’ responses at the PACU and in the postoperative 2nd hour. Afterward follow-up and postoperative demands for analgesics were not evaluated if any delayed hyperalgesia developed. Koppert et al. [30] reported that shortly after cessation of the remifentanil infusion, NRS significantly exceeded control values. Additionally, Tröster et al. [31] reported that RIH developed in the PACU shortly after cessation of remifentanil infusion. Meanwhile, RIH may be relevant to the first postoperative hour in a clinical setting [6]. Almost all RIH developed in the PACU shortly after cessation of remifentanil infusion [31]. The duration and magnitude may be different from prolonged administration periods, high infusion rates, or large doses of remifentanil [26]. As a result, we only followed-up in the first two hours after surgery in our study. Further investigations which follow up to 24 h postoperatively may be advised. Fourth, the analgesic effects of drip infusion of remifentanil 30 min seemed to last for at least 2 h postoperatively, since our analysis revealed significant less NRS in the postoperative 2nd hour in the GWDR group than in the GWR group, which might be related to the analgesic effect of remifentanil. Nevertheless, Koppert et al. [30] showed that remifentanil significantly decreased pain ratings and puncture hyperalgesia only during the infusion period compared with the control group. In addition, shortly after cessation of the infusion, both pain ratings and areas of puncture hyperalgesia exceeded control values and this anti-analgesic effect was most prominent at 30 min after cessation of the infusion. Thereafter, pain ratings gradually declined but remained elevated compared with the control values. They concluded that remifentanil reduced pain (analgesia) and areas of puncture hyperalgesia (anti-hyperalgesia) only during infusion. Our strategy of gradual withdrawal 10–20 min before the end of the surgery followed by drip-infusion for 30 min was intended to extend the analgesic effect of remifentanil and attenuate the development of RIH, as an expanding investigation of a previous study by Saxena et al. [7]. Finally, the multimodal strategy of analgesia is effective for postoperative surgical pain. In our practice, ketorolac was prescribed if no contraindications could have created a confounding bias in our study.

## 5. Conclusions

We first reported that patients receiving gradual withdrawal combined with drip infusion of remifentanil had significantly less rescue analgesics requirement without adverse effects and lower pain scores than those with only gradual withdrawal. This is a unique and a new method for the administration of remifentanil and may be an alternative strategy of gradual withdrawal that can efficiently decrease postoperative pain intensity and analgesic demand to prevent RIH.

## Figures and Tables

**Table 1:** Demographic data.

	Group GWDR (*n* = 205)	Group GWR (*n* = 354)	*p*
Baseline data			
Age (per year)	52.7 ± 17.2	54.9 ± 16.4	0.254
Gender			0.019
Male	93 (45.4)	124 (35.0)	
Female	112 (54.6)	230 (65.0)	
Weight (kg)	64.8 ± 14.3	63.8 ± 14.1	0.633
BMI	24.3 ± 4.7	24.9 ± 10.5	0.451
BMI			0.152
Normal weight (18.5–24)	102 (49.8)	156 (44.1)	
Underweight (<18.5)	9 (4.4)	29 (8.2)	
Overweight (≥24)	94 (45.9)	169 (47.7)	
Intraoperative data			
Type of Surgery			<0.001
Minor ^a^	104 (50.7)	237 (66.9)	
Major ^b^	101 (49.3)	117 (33.1)	
Surgical site			<0.001
Skin and connective tissue	71 (34.6)	66 (18.6)	
Musculoskeletal	14 (6.8)	20 (5.6)	
Chest	72 (35.1)	85 (24.0)	
Abdominal	48 (23.4)	183 (51.7)	
Prescription of postoperative rescue analgesic	27 (13.2)	126 (35.7)	<0.001
Anesthesia time (min)	146 ± 94.8	131 ± 83.6	0.022
Surgical time (min)	117 ± 86.1	103 ± 75.0	0.015
Propofol consumption (mL)	85.1 ± 65.1	76.1 ± 47.8	0.008
Remifentanil consumption (mcg)	529 ± 441	471 ± 363	0.433
Outcome			
NRS at the PACU	2.03 ± 1.85	2.81 ± 1.91	<0.001
NRS group at the PACU			<0.001
0	54 (26.3)	62 (17.5)	
1–3	118 (57.6)	163 (46.0)	
≥4	33 (16.1)	129 (36.4)	
NRS in the postoperative 2nd hour ^c^	2.72 ± 1.03	3.07 ± 1.25	0.001
NRS group in the postoperative 2nd hour ^c^			<0.001
0	2 (1.0)	4 (1.2)	
1–3	149 (78.0)	176 (53.2)	
≥4	40 (20.9)	151 (45.6)	

GWDR = gradual withdrawal combined with drip-infusion of remifentanil; GWR = gradual withdrawal of remifentanil; BMI = body mass index; NRS = numeric rating scale. ^a^ Minor surgery was defined as laparoscopy, arthroscopy, ophthalmic surgery, procedure in skin or mucus membranes and connective tissue. ^b^ Major surgery was defined as musculoskeletal, thoracic, and abdominal surgeries. ^c^ Because of missing medical records, *n* = 191 in the GWDR group and *n* = 331 in the GWR group.

**Table 2:** Logistic regression analysis demonstrating factors associated with low or high NRS (ref: NRS = 0).

	Mild Pain (NRS 1–3/10) (ref: NRS = 0)				Moderate to Severe Pain (NRS ≥ 4/10) (ref: NRS = 0)			
	Univariate Analysis		Multivariable Analysis		Univariate Analysis		Multivariable Analysis	
	OR (95% CI)	*p*	OR (95% CI)	*p*	OR (95% CI)	*p*	OR (95% CI)	*p*
Drip-infusion of Remifentanil (ref: no)	0.83 (0.54–1.28)	0.405	0.30 (0.17–0.55)	<0.001	0.29 (0.17–0.50)	<0.001	0.11 (0.05–0.24)	<0.001
Type of surgery (ref: Major)	0.26 (0.15–0.47)	<0.001	0.34 (0.15–0.79)	0.012	0.11 (0.06–0.20)	<0.001	0.30 (0.11–0.80)	0.016
Surgical site (ref: skin and connective tissue)								
Musculoskeletal	4.56 (2.38–8.75)	<0.001	5.09 (2.14–12.1)	<0.001	7.80 (3.75–16.2)	<0.001	10.9 (3.66–32.2)	<0.001
Chest	1.33 (0.43–4.13)	0.617	1.11 (0.26–4.74)	0.883	9.22 (3.21–26.5)	<0.001	4.88 (1.01–23.5)	0.048
Abdominal	3.95 (2.13–7.35)	<0.001	5.03 (2.30–11.0)	<0.001	9.53 (4.80–18.9)	<0.001	10.9 (3.88–30.4)	<0.001
Age (per year)	0.99 (0.98–1.01)	0.592			1.00 (0.99–1.01)	0.996		
Male	0.75 (0.48–1.17)	0.205			1.27 (0.78–2.05)	0.339		
Weight (kg)	1.00 (0.99–1.02)	0.837			0.99 (0.98–1.01)	0.611		
BMI (ref: normal weight)								
Underweight	0.73 (0.31–1.77)	0.489			0.78 (0.31–1.94)	0.592		
Overweight	1.06 (0.68–1.67)	0.793			0.84 (0.51–1.39)	0.500		
Anesthesia time (per hour)	1.31 (1.08–1.59)	0.005			1.68 (1.32–2.12)	<0.001		
Surgical time (per hour)	1.34 (1.08–1.65)	0.008			1.71 (1.32–2.22)	<0.001		
Propofol consumption (per 100 mL)	2.41 (1.34–4.34)	0.003			5.04 (2.37–10.7)	<0.001		
Remifentanil consumption (per 100 mcg)	1.11 (1.03–1.20)	0.006			1.18 (1.08–1.28)	<0.001		

Variables were selected by forward-selection methods in multivariable analysis.

**Table 3:** Linear regression analysis demonstrating factors associated with elevated NRS.

	NRS at the PACU				NRS in the Postoperative 2nd h			
	Univariate Analysis		Multivariable Analysis		Univariate Analysis		Multivariable Analysis	
	Beta (95% CI)	*p*	Beta (95% CI)	*p*	Beta (95% CI)	*p*	Beta (95% CI)	*p*
Drip infusion of remifentanil (ref: no)	−0.77 (−1.10–−0.45)	<0.001	−1.20 (−1.51–−0.89)	<0.001	−0.35 (−0.56–−0.14)	0.001	−0.31 (−0.52–−0.11)	0.003
Type of Surgery (ref: major)	−1.17 (−1.48–−0.85)	<0.001	−0.55 (−0.94–−0.16)	0.006	0.06 (−0.15–0.27)	0.555		
Surgery site								
skin and connective tissue	−1.26 (−1.57–−0.96)	<0.001	−1.24 (−1.64–−0.85)	<0.001	0.19 (−0.02–0.40)	0.073		
Musculoskeletal	0.39 (0.02–0.76)	0.039			−0.36 (−0.60–−0.13)	0.003		
Chest	1.07 (0.41–1.73)	0.002			−0.52 (−0.95–−0.09)	0.018		
Abdominal	0.86 (0.51–1.21)	<0.001			0.24 (0.02–0.47)	0.035	0.27 (0.05–0.48)	0.016
Age (per year)	−0.01 (−0.01–0.01)	0.888			0.01 (−0.01–0.01)	0.156		
Female	−0.22 (−0.55–0.11)	0.183			0.74 (0.54–0.94)	<0.001	0.70 (0.50–0.90)	<0.001
Weight (kg)	−0.06 (−0.17–0.05)	0.286			−0.10 (−0.18–−0.03)	0.004		
BMI (ref:)								
Normal weight	0.13 (−0.19–0.45)	0.427			−0.08 (−0.28–0.13)	0.474		
Underweight	−0.01 (−0.65–0.63)	0.974			−0.19 (−0.22–0.59)	0.370		
Overweight	−0.13 (−0.45–0.19)	0.437			0.03 (−0.18–0.23)	0.790		
Anesthesia time (per hour)	0.21 (0.11–0.32)	<0.001			−0.10 (−0.17–−0.03)	0.004		
Surgical time (per hour)	0.23 (0.11–0.35)	<0.001			−0.10 (−0.17–−0.02)	0.012		
Propofol consumption (per 100 mL)	0.41 (0.12–0.70)	0.006			−0.17 (−0.36–0.01)	0.063		
Remifentanil consumption (per 100 mcg)	0.07 (0.03–0.11)	<0.001			−0.03 (−0.06–−0.01)	0.011		

Variables in the multivariable analysis were selected by stepwise method.

**Table 4:** Logistic regression analysis demonstrating factors associated with use of rescue analgesics (ref: no use).

	Univariate Analysis		Multivariable Analysis	
	OR (95% C.I.)	*p*	OR (95% C.I.)	*p*
Drip infusion of Remifentanil (ref: no)	0.27 (0.17–0.43)	<0.001	0.14 (0.08–0.24)	<0.001
Type of surgery (ref: Major)	0.26 (0.18–0.39)	<0.001	0.43 (0.24–0.75)	0.003
Surgical site (ref: skin and connective tissue)				
Musculoskeletal	2.85 (1.69–4.82)	<0.001	3.24 (1.62–6.51)	0.001
Chest	10.4 (4.74–22.9)	<0.001	9.86 (3.41–28.5)	<0.001
Abdominal	3.92 (2.39–6.46)	<0.001	4.27 (2.24–8.14)	<0.001
Age (per year)	1.00 (0.99–1.01)	0.648		
Male	1.37 (0.94–2.01)	0.099		
Weight (per 10 kg)	0.90 (0.79–1.04)	0.148	0.84 (0.72–0.99)	0.035
BMI (ref: normal weight)				
Underweight	1.30 (0.63–2.68)	0.483		
Overweight	0.77 (0.52–1.14)	0.188		
Anesthesia time (per hour)	1.22 (1.08–1.37)	0.001		
Surgical time (per hour)	1.24 (1.08–1.41)	0.002		
Propofol consumption (per 100 mL)	1.35 (0.98–1.86)	0.066		
Remifentanil consumption (per 100 mcg)	1.06 (1.01–1.11)	0.011		

Variables were selected by forward-selection methods in multivariable analysis.

## Data Availability

The data presented in this study are available on request from the corresponding author.
